# Bi-level CPAP does not change central blood flow in preterm infants with respiratory distress syndrome

**DOI:** 10.1186/1824-7288-40-60

**Published:** 2014-06-21

**Authors:** Giulia Aquilano, Silvia Galletti, Arianna Aceti, Francesca Vitali, Giacomo Faldella

**Affiliations:** 1 Neonatology and Neonatal Intensive Care Unit, Department of Medical and Surgical Sciences, St. Orsola-Malpighi Hospital - University of Bologna, Via Massarenti, 11 40138 Bologna, Italy

## Abstract

**Background:**

Current literature provides limited data on the hemodynamic changes that may occur during bi-level continuous positive airway pressure (CPAP) support in preterm infants. However, the application of a positive end-expiratory pressure may be transmitted to the heart and the great vessels resulting in changes of central blood flow.

**Objective:**

To assess changes in central blood flow in infants with respiratory distress syndrome (RDS) during bi-level CPAP support.

**Design:**

A prospective study was performed in a cohort of 18 Very-Low-Birth-Weight Infants who were put on nasal CPAP support (4–5 cmH_2_O) because they developed RDS within the first 24–72 hours of life. Each subject was switched to bi-level CPAP support (Phigh 8 cmH_2_O, Plow 4–5 cmH_2_O, Thigh 0.5-0.6 seconds, 20 breaths/min) for an hour. An echocardiographic study and a capillary gas analysis were performed before and after the change of respiratory support.

**Results:**

No differences between n-CPAP and bi-level CPAP in left ventricular output (LVO, 222.17 ± 81.4 vs 211.4 ± 75.3 ml/kg/min), right ventricular output (RVO, 287.8 ± 96 vs 283.4 ± 87.4 ml/kg/min) and superior vena cava flow (SVC, 135.38 ± 47.8 vs 137.48 ± 46.6 ml/kg/min) were observed. The hemodynamic characteristics of the ductus arteriosus were similar. A significant decrease in pCO_2_ levels after bi-level CPAP ventilation was observed; pCO_2_ variations did not correlate with modifications of central blood flow (LVO: ρ = 0.11, p = 0,657; RVO: ρ = −0.307, p = 0.216; SVC: ρ = −0.13, p = 0.197).

**Conclusions:**

Central blood flow doesn’t change during bi-level CPAP support, which could become a hemodinamically safe tool for the treatment of RDS in preterm infants.

## Introduction

Bi-level CPAP is a form of respiratory support routinely used to assist adults and children. It enables the patient to breathe spontaneously between two different levels of continuous positive pressure, the upper (IPAP) and the lower level (EPAP). The cycle time can be either selected by the operator or synchronized with the patient’s respiratory effort. Both IPAP and EPAP are generated by an increase in the gas flow through the circuit without closing the expiratory valve enabling the patient to exhale anytime, even during the IPAP phase [1]. The aim of bi-level CPAP is to improve alveolar recruitment, gas exchange and, by a direct stimulation of the upper airways, to prevent apneas requiring intubation.

Nasal CPAP (n-CPAP) is currently considered the treatment of choice for neonatal respiratory distress syndrome (RDS) [2]. By applying a constant distending pressure to the airways n-CPAP maintains the functional residual capacity (FRC) of the lungs through alveolar recruitment and stabilization. This constant pressure prevents alveolar collapse during expiration, improves gas exchange and reduces the work of breathing facilitating the next inspiratory phase [3].

The theoretical advantage of bi-level CPAP could be the prevention of alveolar collapse through a swing between two different FRC. Furthermore the change in pressure (ΔP) created by this mechanism could generate a tidal volume responsible for a decrease in the work of breathing [4].

However only few studies were performed on newborns and they were mainly focused on term babies with central hypoventilation syndrome [5].

To date there are few published data on the effects of bi-level CPAP in preterm infants and no data on the hemodynamic changes related to the use of this respiratory support. Nevertheless, it has been demonstrated that the application of an end-expiratory positive pressure (PEEP) can interfere with venous return and cardiac output [6].

The present study aims to evaluate the hemodynamic effects on cardiac outputs and venous return of bi-level CPAP in a population of Very Low Birth Weight Infants (VLBWIs) with RDS.

## Methods

### Patients

A prospective study was conducted in a cohort of VLBWIs admitted at birth to the Neonatal Intensive Care Unit (NICU) of Bologna University Hospital (Italy) between January 2012 and May 2012. Infants were eligible for inclusion in the study if they developed signs of RDS requiring nCPAP support within the first 24–72 hours of life. RDS was defined in presence of clinical features of respiratory distress (tachypnea, grunting, chest retractions, nasal flaring), need of continuous positive pressure to maintain oxygen saturation > 85% and compatible radiological findings (e.g. reticulo-granular pattern, ground glass appearance, air bronchograms, reduced lung volume).

In order to be included in the study, infants should have been hemodynamically stable (normal blood pressure, normal urine output and no need for inotropes).

Infants with congenital heart disease, congenital malformations or assisted with mechanical ventilation were excluded.

Each patient was supported with n-CPAP for several hours, and switched to bi-level CPAP for a study period of an hour. At the end of the study period all the subjects were put back on n-CPAP.

The Infant Flow**®** SiPAP system (Viasys Healthcare, Yorba Linda, CA, USA), which allows the operator to switch from n-CPAP to bi-level CPAP mode without interruption of the respiratory support or changes of the device circuit, was used for the present study. Short bi-nasal prongs of the largest size fitting comfortably the patient’s nares were used in order to minimize air leaks. Alternatively, nasal masks of the appropriate size for the patient’s nose were used.

Nasal CPAP was set at 4–5 cm H20 and bi-level CPAP as follows: Phigh 8 cm H_2_O, Plow 4-5cmH_2_O, Thigh 0.5-0.6 seconds, rate 20 breaths/min. The minimal fractional inspired oxygen saturation (FiO_2_) was set in order to maintain oxygen saturation (SpO2), monitored through a transcutaneous device, between 85 and 94%. Before and after the study period, blood pressure was recorded, a capillary blood gas analysis was collected to detect the PCO_2_ level, and an echocardiographic study was performed to evaluate the hemodynamic parameters.

Blood pressure was measured non-invasively by the oscillometric method. Three values of mean blood pressure were averaged and compared with normal values for gestational age.

### Hemodynamic measurements

The echocardiographic study was performed using a Philips HD11 XE (Philips Ultrasound, Andover, MA, USA) ultrasound system with a 12-4 Hz transducer incorporating colour flow and pulse wave Doppler. No angle correction was used. Left ventricular output (LVO), right ventricular output (RVO), superior vena cava (SVC) flow, the diameter and the characteristics of the *ductus arteriosus* (DA) and *foramen ovale* were evaluated. RVO, LVO and SVC flow were measured according to previously published methodology [7,8]. For the calculation of flow, the following formula was used: flow = (velocity time integral × (π × (diameter2/4) × heart rate)/body weight. Six or more cycles were averaged for these measurements. Cardiac outputs <150/kg/min and SVC flow <40 ml/kg/min were considered as low blood flows [7,8]. Ductal diameter was determined from a 2D image, using a high parasternal position in a way that the whole length of the duct could be visualized. The diameter was taken at the smallest point at the pulmonary side of the duct. Maximum LR flow velocity (Vmax), flow pattern (continuous, pulsatile) and % of RL shunt were also recorded [9]. The McNamara echocardiographic criteria were used to define an hemodynamically significant duct [10]. The presence of a patent *foramen ovale* was also assessed and a diameter >3 mm was considered hemodynamically significant. For each patient, all echocardiographic measurements were taken by a single operator. All the images were saved on magnetic optical disks. The EchoPAC software (GE Vingmed Ultrasound EchoPAC 7.00, Horten, Norway) was used for off-line analysis performed by a second investigator blinded to the respiratory support method. Ethics approval and written informed consent were obtained from each patient parent/guardian. The study protocol was approved by the Institutional Ethics Committee of S.Orsola-Malpighi Hospital, Bologna.

### Statistics

Statistical analysis was performed using SPSS 13.0 for Windows (Statistical Package for Social Sciences, SPSS Inc, Chicago, Ill, US).

A preliminary analysis was made to calculate the power of the study and a type II error probability. Based on previous studies in literature [11] we estimated that a sample size of 16 patients was sufficient to observe a 25% reduction of both cardiac outputs (power = 0.8, α = 0.05, one tail).

Comparison of echocardiographic measurements between n-CPAP and bi-level CPAP was performed using Wilcoxon signed rank test. Spearman test was used to correlate RVO and LVO with SVC flow. Results were expressed as mean ± standard deviation if not specified otherwise. P values <0.05 were considered statistically significant.

## Results

Thirty-two VLBWIs were admitted to the NICU during the study period. Fourteen subjects (43.8%) were excluded for the following reasons: 2 infants were on mechanical ventilation, 6 without respiratory support or with nasal cannulae, 2 infants had congenital heart disease and 4 were not enrolled because the investigator was unavailable at the time of the study.

The demographic characteristics of the enrolled infants are reported in Table 1.

**Table 1:** Demographic characteristics of the study population

**Patients**	**Sex**	**GA (wks + days)**	**BW(g)**	**Surfactant**	**FiO**_**2**_	**Age at the time of the study (h)**
1	M	33 + 3	1080	Yes	30	25
2	M	28	1230	Yes	21	18
3	F	25 + 6	934	Yes	21	28
4	M	30	1320	Yes	21	23
5	M	29 + 6	1147	No	21	32
6	F	32 + 2	1407	No	21	16
7	F	28	990	Yes	21	42
8	F	26	757	Yes	25	55
9	M	30	1322	Yes	21	30
10	F	33 + 4	1447	No	21	9
11	F	30	880	Yes	25	61
12	F	30	499	Yes	30	65
13	M	26 + 4	700	No	25	37
14	F	32	1076	No	21	35
15	M	33	1060	No	25	24
16	F	32 + 1	1490	No	21	10
17	M	27 + 5	1076	No	21	36
18	F	27 + 5	915	No	25	36

GA, gestational age; BW birth weight.

The 18 subjects included in the study (8 males, 10 females) had a median gestational age (GA) of 30 weeks (range 25–33 weeks) and a median birth weight (BW) of 1076 g (range 499–1490 g). All infants were assisted with n-CPAP because of respiratory distress; surfactant was administered to 50% of the subjects prior the enrollment in the present study. The subjects received surfactant either on admission to the unit when intubated at birth in the delivery room, or within 4 hours from birth following the InSurE (Intubation-Surfactant-Extubation) procedure when not intubated at birth (age at the time of surfactant administration: 0–4 hours of life). For these subjects the study was performed at least 12 hours after surfactant administration (age at the time of the study: 18–65 hours of life).

Mean vascular diameters were: aorta 0.47 cm ±0.06, pulmonary artery 0.5 cm ± 0.05, SVC 0.33 cm ± 0.07. Fourteen newborns (77.8%) had a PDA at the time of the study. Eight subjects met the echocardiographic criteria for a moderate hemodynamically significant duct.

Echocardiographic measurements are reported in Table 2: there were no significant changes of blood flow between the two different methods of respiratory support. However, a significant reduction of pCO2 values was observed after the respiratory support with the bi-level CPAP (p = 0.01).

**Table 2:** Comparison between hemodynamic parameters: bi-level vs nasal CPAP (mean ± SD)

	**nCPAP**	**Bilevel**	**p**
**LVO (ml/kg/min)**	222.17 ± 81.4	211.4 ± 75.3	.35
VTI (cm)	8.25 ± 2	7.93 ± 1.3	.42
SV (ml)	1.49 ± 0.6	1.41 ± 0.4	.35
**RVO (ml/kg)min)**	287.8 ± 96	283.4 ± 87.4	.71
VTI (cm)	10.17 ± 2.8	9.86 ± 1.8	.56
SV (ml)	1.97 ± 0.7	1.96 ± 0.6	.85
**SVC (ml/kg/min)**	135.38 ± 47.8	137.48 ± 46.6	.50
VTI (cm)	11.3 ± 3.2	11.87 ± 2.9	.35
HR (bpm)	146.88 ± 2.07	143.55 ± 1.56	.61
pCO2 (mm Hg)	47.96 ± 6.9	43.66 ± 4.5	.01

Four patients presented LVO values <150 ml/kg/min both on nCPAP and bi-level CPAP; moreover, 2 patients had a low LVO only on bi-level CPAP. No low RVO or SVC flow was observed. No differences were also observed when considering the hemodynamic characteristics of the DA (Table 3).

**Table 3:** Hemodynamic characteristics of the ductus arteriosus (mean ± SD)

	**nCPAP**	**Bilevel**	**p**
**Ductus arteriosus**			
Pulsatile pattern (%)	57.1	57.1	n/a
Vmax (m/sec)	1.68 ± 0.5	1.46 ± 0.38	0.19
% R-L shunt	6.2 ± 10.6	12.6 ± 11.7	0.22

Significant positive correlations between LVO and SVC flow were found both in nasal (ρ = 0.82; p = 0.00) and bi-level CPAP (ρ = 0.6; p = 0.00) while a positive correlation between RVO and SVC flow was present when the patients where on n-CPAP (ρ = 0.73; p = 0.01) but not on bi-level CPAP (ρ = 0.37; p = 0.14). When comparing the characteristics of the subjects that showed an improvement in central blood flows on bi-level CPAP with those who showed a worsening no significant differences were found in terms of BW and GA. Moreover, pCO_2_ variations did not correlate with modifications of central blood flow (LVO: ρ = 0.11, p = 0,657; RVO: ρ = −0.307, p = 0.216; SVC: ρ = −0.13, p = 0.197).

## Discussion

The present study shows that bi-level CPAP does not affect central blood flow in preterm infants with RDS.

Current literature regarding the application of bi-level CPAP to infants is scarce and mainly focused on its efficacy as a non-invasive ventilation tool [4,12,13].

To date few papers were published on preterm newborns. One of these studies showed a significant gas exchange improvement when the babies were assisted with bi-level CPAP [10]. In a randomized controlled trial (RCT) that compared the use of nasal versus bi-level CPAP in infants with RDS, the subjects randomized in the bi-level branch showed a better outcome in terms of days of respiratory support and days of oxygen dependency [4].

The effectiveness of bi-level CPAP in the post-extubation phase is controversial. The support with bi-level CPAP right after the InSurE procedure seemed to reduce the chances of extubation failure and need of mechanical ventilation [13]; however, a randomized trial published in 2012 showed no differences in the rate of sustained extubation (>7 days) between the subjects assisted with bi-level CPAP and those with nasal CPAP [14].

A recent trial showed that nasal intermittent positive-pressure ventilation (IPPV) is not superior to nasal CPAP in terms of major outcomes such as death and bronchopulmonary dysplasia [15].

To our knowledge this is the first study that has evaluated the safety of bi-level CPAP from the hemodynamic point of view.

Interactions between respiratory and cardiovascular systems are complex; in particular the application of an end-expiratory positive pressure (PEEP) changes these interactions so that on one hand oxygenation is improved but on the other it can interfere with venous return and therefore with cardiac output [16].

Animal model studies show that PEEP modifies the shape of venous return curve by increasing the critical pressure through a distortion of venous geometry, for example at the entrance of the venae cavae into the thorax. It is important to note that volemic status may largely influence the effects of PEEP. In fact hypovolaemia is likely to blunt the compensatory rise in systemic blood pressure; conversely, volemic expansion can contrast the effect of PEEP on venous return [6].

Theoretically there is potential for higher PEEP, as by opening up the lungs, it may reduce pulmonary vascular resistance and increase pulmonary and systemic blood flow [11].

Several studies focused on the cardiovascular effects of PEEP administration through invasive and non-invasive mechanical ventilation. Nasal CPAP with a mean level of 4 cm H_2_O does not influence cardiac output in preterm infants [17]; however a study on animal models showed that increases of PEEP from 4 to 10 cm H_2_O led to a significant decrease in the left pulmonary artery flow and an increased right to left shunting through the *ductus arteriosus*; the authors speculated this was likely due to the concurrent increase in the pulmonary vascular resistance and to a physical constraint (increasing intrathoracic pressure) placed on the heart by the over-expanded lung [18].

In a study on mechanically ventilated newborns, an increase of PEEP from 5 to 8 cmH_2_O led to a RVO decrease without causing either positive or negative relevant clinical effects on systemic blood flow in the majority of the infants [11]. However 36% of the subjects showed an improvement in SVC flow presumably as a consequence of improved lung compliance due to alveolar recruitment and an optimised pulmonary volume. When PEEP is set too low, blood will be shunted away from the collapsed alveoli producing a regional increase in pulmonary vascular resistance.

The present study demonstrated that bi-level CPAP does not modify venous return and cardiac outputs.

In this study RVO and LVO mean values were consistent with those reported in literature [7] while mean SVC values resulted slightly higher [8]. This difference may be ascribed to the higher complexity of SVC flow echocardiographic measurement. Both SVC diameters and doppler velocities resulted higher than the values reported in literature. However, if any error was made in the assessment of the SVC flow, it is unlikely that it could have altered the comparison between the two ventilation modalities because the same operator performed both the echocardiographic assessments of each patient. Overall 6 infants showed low LVO without any clinical implication; in fact all the enrolled subjects remained stable (normal blood pressure, normal urine output, no need for inotropes) during the whole study period.

A recent study [19] performed on preterm newborns in the first hour of life showed a LVO well below the threshold of 150 ml/kg/min suggested by Evans et al. [7] for the first 24 hours of life. The authors found a mean LVO value of 71 ml/kg/min (range 61–110 at 1 hour of life and ascribed this finding to the progressive physiological improvement of cardiac function during the transitional period [20]. This might explain our findings.

While cardiac outputs are greatly influenced by both ductal and atrial shunting during the transitional period [21,22] SVC flow is independent [20] resulting in a lack of relationship between cardiac outputs and SVC flow [21]. On the opposite, we found significant positive correlations between outputs and SVC flow. Only the correlation between RVO and SVC during bi-level CPAP support resulted to be still positive but not statistically significant.

We hypothesized that atrial and ductal shunting did not represent a variable in our study. In fact the characteristics of the *ductus arteriosus* (flow pattern, velocity peak,% of left-to-right shunt) were similar during bi-level and nasal NCPAP support. Moreover a significant ductal shunt results in an increased LVO to RVO ratio due to a relative increase of LVO [9], a finding that we did not observe in our study.

We found a significant decrease in pCO2 during bi-level CPAP support that was not related to central blood flow variations and thus not to be ascribed to a change in the pulmonary perfusion. It’s likely that bi-level CPAP, by cyclic variations of PEEP, may improve alveolar recruitment and optimize ventilation resulting in a decrease of pCO2 as described by previous studies [12]. However because the decrease in pCO2 is not clinically relevant (47.96 ± 6.9 vs 43.66 ± 4.5 mmHg) we would not speculate further.

The demographic characteristics of our population were analysed in order to detect differences between the subjects who decreased central blood flow during bi-level CPAP support and those who didn’t and no differences were found in terms of GA and BW.

Therefore on the basis of these findings it is not possible to identify a group of subjects that may specifically benefit from one of the two ventilatory support.

A paper published in 2009 [4] found that bi-level CPAP, when used as a first choice treatment for RDS in preterm babies, induced the same changes of nasal CPAP in the level pulmonary inflammatory cytokines.

The present study adds that bi-level CPAP is a safe ventilatory choice not only in terms of pulmonary injury but also from the hemodynamic point of view.

The main limitations of the study are the small number of patients included and the selective inclusion criteria.

The sample size was adequate to observe clinically relevant changes in blood flow; however it’s not sufficient to exclude flow changes less than 25%. Such small changes in central blood flow may not have clinical implications. In our study the application of bi-level CPAP resulted in small changes (sometimes improvement, sometimes worsening) of central blood flow and this is probably why changes in the clinical conditions of the patients were not observed.

Moreover echocardiography is a technique with many weaknesses: measurement errors, poor image quality due to a limited acoustic access in some patients, artefacts. All these limitations play an important role and should be taken into account especially when the number of the patients is small such as in the present study.

Lastly, we included in the study only stable infants without cardiovascular compromise and therefore our results may not be extended to critical infants.

In a recent paper, published by Becker et al [23]. the authors measured central blood flow during respiratory support different levels of CPAP (4, 6 and 8 cmH_2_O) in a population of preterms with minimal lung disease. They hypothesized that stable infants (without inotropic support) may be able to compensate for the increased intrathoracic pressure caused by CPAP, that is, they may have had an initial decrease in cardiac output but they might have compensated rapidly with a transient increase in heart rate and subsequent recovery of cardiac output. This may also apply to our findings since the populations and the pressure levels were similar even though we used a different respiratory device. Therefore our results should be interpreted with caution as they may not apply to infants with cardiovascular instability. Other studies should be carried out to confirm our findings in critically ill infants.

## Competing interests

All the authors declare that they have no conflict of interest with this paper.

## Authors’ contributions

GA, SG and GF designed the study. GA performed ECHO scan. FV performed the offline analysis. AA performed statistical analysis. GA and AA wrote the first draft of the paper, which was revised by all the other authors of the paper. All authors read and approved the final manuscript.

## References

[B1] Antonescu-Turcu A Parthasarathy S CPAP and bi-level PAP therapy: new and established roles Respir Care 2010 55 9 1216 1229 20800002 PMC3119924

[B2] Verder H Nasal CPAP has become an indispensable part of the primary treatment of newborns with respiratory distress syndrome Acta Pediatr 2007 96 4 272 276 10.1111/j.1651-2227.2007.00263.x 17391463

[B3] DiBlasi CPAP for respiratory care of the newborn infant Respir Care 2009 54 9 1209 1235 19712498

[B4] Lista G Castoldi F Fontana P Daniele I Caviglioli F Rossi S Mancuso D Reali R Nasal continuous positive airway pressure (CPAP) versus bi-level nasal CPAP in preterm babies with respiratory distress syndrome: a randomised control trial Arch Dis Child Fetal Neonatal Ed 2010 95 F85 F89 19948523 10.1136/adc.2009.169219

[B5] Tibballs J Henning RD Non invasive ventilation strategies in the management of a newborn infant and three children with congenital central hypoventilation syndrome Pediatr Pulmonol 2003 36 544 548 14618648 10.1002/ppul.10392

[B6] Feihl F Broccard AF Interaction between respiration and systemic hemodynamics. Part I: basic concepts Intensive Care Med 2009 35 45 54 18825367 10.1007/s00134-008-1297-z

[B7] Evans N Kluckow M Early determinants of right and left ventricular output in ventilated preterm infants Arch Dis Child 1996 74 F88 F94 10.1136/fn.74.2.f88 PMC2528520 8777673

[B8] Kluckow M Evans N Superior vena cava flow in newborn infants: a novel marker of systemic blood flow Arch Dis Child Fetal Neonatal Ed 2000 82 F182 F187 10794783 10.1136/fn.82.3.F182 PMC1721083

[B9] De Waal K The methodology of doppler derived central blood flow measurement in newborn infants Int J Pediatr 2012 2012 680162 22291718 10.1155/2012/680162 PMC3265082

[B10] McNamara PJ Sehgal A Towards rational management of the patent ductus arteriosus: the need for disease staging Arch Dis Child Fetal Neonatal Ed 2007 92 6 F424 F427 17951547 10.1136/adc.2007.118117 PMC2675381

[B11] De Waal K Evans N Osborn DA Kluckow M Cardiorespiratory effects of changes in end expiratory pressure in ventilated newborns Arch Dis Child Fetal Neonatal Ed 2007 92 F444 F448 17460022 10.1136/adc.2006.103929 PMC2675387

[B12] Migliori C Motta M Angeli A Chirico G Nasal bilevel vs Continuous Positive Airway Pressure in Preterm infants Pediatr Pulmonol 2005 40 426 430 16155882 10.1002/ppul.20276

[B13] Ancora G Maranella E Grandi S Pierantoni L Guglielmi M Faldella G Role of bilevel positive airway pressure in the management of preterm newborns who have received surfactant Acta Pediatr 2010 99 1807 1811 10.1111/j.1651-2227.2010.01910.x 20545934

[B14] O’Brien K Campbell C Brown L Wenger L Shah V Infant flow biphasic nasal continuous positive airway pressure (BP- NCPAP) vs. infant flow NCPAP for the facilitation of extubation in infants ≤ 1,250 grams: a randomized controlled trial BMC Pediatr 2012 12 43 22475409 10.1186/1471-2431-12-43 PMC3402979

[B15] Kirpalani H Millar D Lemyre B Yoder BA Chiu A Roberts RS A Trial Comparing Noninvasive Ventilation Strategies in Preterm Infants N Engl J Med 2013 369 611 620 23944299 10.1056/NEJMoa1214533

[B16] Abdel-Hady H Matter M Hammad A El-Refaay A Aly H Hemodynamic changes during weaning from nasal continuous positive airway pressure Pediatrics 2008 122 5 e1086-90 18977958 10.1542/peds.2008-1193

[B17] Moritz B Fritz M Mann C Simma B Nasal conti does not change cardiac output in preterm infants Am J Perinatol 2008 25 105 110 18240105 10.1055/s-2008-1040341

[B18] Polglase GR Hooper SB Gill AW Allison BJ McLean CJ Nitsos I Pillon JJ Kluckow M Cardiovascular and pulmonary consequences of airway recruitment in preterm lambs J Appl Physiol 2009 106 1347 1355 19213936 10.1152/japplphysiol.91445.2008

[B19] Sehgal A Mak W Dunn E Whyte H McCrindle B McNamara PJ Haemodynamic changes after delivery room surfactant administration to very low birth weight infants Arch Dis Child Fetal Neonatal Ed 2010 95 F345 F351 20538711 10.1136/adc.2009.173724

[B20] Kluckow M Low systemic blood flow and pathophysiology of the preterm transitional circulation Early Hum Dev 2005 81 429 437 15935920 10.1016/j.earlhumdev.2005.03.006

[B21] Miletin J Dempsey EM Low superior vena cava flow on day 1 and adverse out come in the very low birthweight infant Arch Dis Child Fetal Neonatal Ed 2008 93 F368 F371 18089627 10.1136/adc.2007.129304

[B22] Kluckow M Evans N Low systemic blood flow in the preterm infant Semin Neonatol 2001 6 75 84 11162287 10.1053/siny.2000.0035

[B23] Beker F Rogerson SR Hooper SB Wong C Davis PG The effects of nasal continuous positive airway pressure on cardiac function in premature infants with minimal lung disease: a crossover randomized trial J Peds 2014 164 726 729 10.1016/j.jpeds.2013.10.087 24345453

